# Functional parcellation of the hippocampus by semi-supervised clustering of resting state fMRI data

**DOI:** 10.1038/s41598-020-73328-1

**Published:** 2020-10-02

**Authors:** Hewei Cheng, Hancan Zhu, Qiang Zheng, Jie Liu, Guanghua He

**Affiliations:** 1 Department of Biomedical Engineering, School of Bioinformatics, Chongqing University of Posts and Telecommunications, Chongqing, 400065 China; 2 Chongqing Engineering Research Center of Medical Electronics and Information Technology, Chongqing University of Posts and Telecommunications, Chongqing, 400065 China; 3 Chongqing Engineering Laboratory of Digital Medical Equipment and Systems, Chongqing University of Posts and Telecommunications, Chongqing, 400065 China; 4 College of Mathematics Physics and Information, Shaoxing University, Shaoxing, 312000 China; 5 School of Computer and Control Engineering, Yantai University, Yantai, 264005 China; 6 Research Institute of Education Development, Chongqing University of Posts and Telecommunications, Chongqing, 400065 China; 7 College of International Finance and Trade, Zhejiang Yuexiu University of Foreign Languages, Shaoxing, 312000 China

**Keywords:** Neuroscience, Nervous system

## Abstract

Many unsupervised methods are widely used for parcellating the brain. However, unsupervised methods aren’t able to integrate prior information, obtained from such as exiting functional neuroanatomy studies, to parcellate the brain, whereas the prior information guided semi-supervised method can generate more reliable brain parcellation. In this study, we propose a novel semi-supervised clustering method for parcellating the brain into spatially and functionally consistent parcels based on resting state functional magnetic resonance imaging (fMRI) data. Particularly, the prior supervised and spatial information is integrated into spectral clustering to achieve reliable brain parcellation. The proposed method has been validated in the hippocampus parcellation based on resting state fMRI data of 20 healthy adult subjects. The experimental results have demonstrated that the proposed method could successfully parcellate the hippocampus into head, body and tail parcels. The distinctive functional connectivity patterns of these parcels have further demonstrated the validity of the parcellation results. The effects of aging on the three hippocampus parcels’ functional connectivity were also explored across the healthy adult subjects. Compared with state-of-the-art methods, the proposed method had better performance on functional homogeneity. Furthermore, the proposed method had good test–retest reproducibility validated by parcellating the hippocampus based on three repeated resting state fMRI scans from 24 healthy adult subjects.

## Introduction

The hippocampus is comprised of multiple functionally and anatomically heterogeneous subfields, which plays an important role in memory functions. Changes of the hippocampal subfields have been identified in many neuroimaging studies of the normal aging as well as neuropsychiatric diseases, such as Alzheimer’s disease, depression, anxiety disorder, and schizophrenia. Several studies have reported that the hippocampus could be subdivided into multiple anatomically different subregions based on structural magnetic resonance imaging (sMRI) data^1–5^. Additionally, the hippocampus has multiple functionally heterogeneous parcels along its longitudinal axis^2,6,7^, and different parcels have distinguishable memory functions^8–11^. The sMRI based studies have demonstrated that the hippocampus could be parcellated into at least three parcels, i.e., head, body, and tail, along the hippocampal longitudinal axis according to brain landmarks^1–5,7^. However, the structural subregions obtained by using the sMRI data are not necessarily matched with their functions^12^.

Recently, several methods have been presented to parcellate the hippocampus into functionally homogeneous subregions based on functional neuroimaging data, such as independent component analysis (ICA)^13–15^, meta-analytic connectivity modeling^16,17^, consensus clustering based multimodal parcellation^18^, and preferred functional connectivity based parcellation^19,20^. Particularly, the masked ICA based method can parcellate the hippocampus into several parcels along the hippocampal longitudinal axis based on resting state functional magnetic resonance imaging (fMRI) data^13–15^, but the method is not robust enough for subject-specific parcellation. In addition, the meta-analytic method can also parcellate the hippocampus into several parcels along the hippocampal longitudinal axis^16,17^. The method integrates co-occurrence of significant activations across functional neuroimaging studies to generate a single parcellation. The consensus clustering based multimodal parcellation method can functionally divide the hippocampus into head, body, and tail parts along the hippocampal longitudinal axis based on both meta-analytic data and resting state fMRI data^18^. The parcellation obtained by using the method might be biased towards one modality. Furthermore, other studies have demonstrated that the hippocampus could be parcellated into two or three parcels along the hippocampal longitudinal axis according to preferred functional connections with cortical regions^19,20^. The parcellation generated by using the method is dependent on the selected cortical regions. In the current study, we aim to parcellate the hippocampus into subject-specific spatially and functionally consistent parcels using a semi-supervised brain parcellation method with the structural parcellation as the prior information based on resting state fMRI data. Specifically, each subregion of the structural parcellation is subdivided based on each voxel’s functional consistency with its neighboring voxels using watershed segmentation algorithm^21^. Then, one subdivision within each subregion of the structural parcellation that is most functionally homogeneous is chosen as the structural prior label for optimizing functional parcellation in the proposed semi-supervised method.

Besides the aforementioned methods specifically designed for the hippocampus parcellation, a large number of unsupervised clustering based methods, such as spectral clustering^22–30^, hierarchical clustering^31–33^, graph cut based clustering^34–36^, *k*-means^37–43^, have been utilized for functional brain parcellation based on resting state fMRI data. Nevertheless, these unsupervised clustering based brain parcellation methods are not able to integrate prior information that can be obtained from meta-analysis results, cytoarchitectonic parcellation results, or existed functional neuroanatomy studies, although such prior information is frequently adopted for determining the clustering number in neuroimaging brain parcellation studies. More recently, a semi-supervised clustering based brain parcellation method has been presented for parcellating the brain through integrating prior information based on resting state fMRI data in our preliminary studies^44–46^. Compared with unsupervised brain parcellation methods, the semi-supervised clustering based brain parcellation method is not only robust to imaging noise, bust also able to integrate prior information as a supervision for reliable brain parcellation^44–46^.

In this study, we present a semi-supervised clustering based brain parcellation method derived from a semi-supervised graph partitioning algorithm^47,48^, aiming to obtain a spatially and functionally consistent parcellation of the hippocampus with improved performance based on resting state fMRI data. Particularly, the hippocampus parcellation is modeled as a graph partitioning problem. All voxels within the hippocampus are modeled as nodes of an undirected graph, and edges are formed between each pair of these voxels. The weight on each edge is measured by the similarity of their functional signals between voxels. The modeled graph according to our preliminary study is partitioned into disjoint subgraphs by optimizing^45^: (1) similarity of nodes in each subgraph, (2) similarity between the brain partition and prior information, obtained from such as meta-analysis results, cytoarchitectonic parcellation results^45,46^, or existed functional neuroanatomy studies^44,45^, and (3) spatial connectedness of the brain partition^49–51^. The optimal solution of the graph partitioning problem is solved by a weighted kernel *k*-means algorithm^47,52^. The voxels belonging to nodes of each subgraph constitute one parcel. Furthermore, hyper-parameters in the graph partitioning problem are tuned for improving functional homogeneity of the brain partition.

We validated the presented method by applying to 20 healthy adult subjects’ resting state fMRI data in hippocampus parcellation. We further evaluated the test–retest reproducibility of the presented method based on another 25 healthy adult subjects’ resting state fMRI data, each of them having 3 repeated scans. The experimental results have illustrated that the presented method could successfully parcellate the hippocampus into head, body and tail parcels along the hippocampal longitudinal axis^2,19^. The three hippocampus parcels had different functional connectivity patterns, suggesting that the obtained parcels might be functionally meaningful^37,53^. In an application to expore aging effects on functional connectivity, it was found that the increasing age was accompanied by decreased hippocampus parcels’ functional connectivity across the healthy subjects in the adult aging span. Compared with state-of-the-art brain parcellation methods, the presented semi-supervised brain parcellation method had superior performance on functional homogeneity. The results of test–retest reproducibility experiments have illustrated that the presented method could generate reproducible parcellation results of the same brain based on resting state fMRI data scanned at different time points.

## Results

### Validation through hippocampus parcellation

#### Validation through examining parcellation results

**Figure 1:**
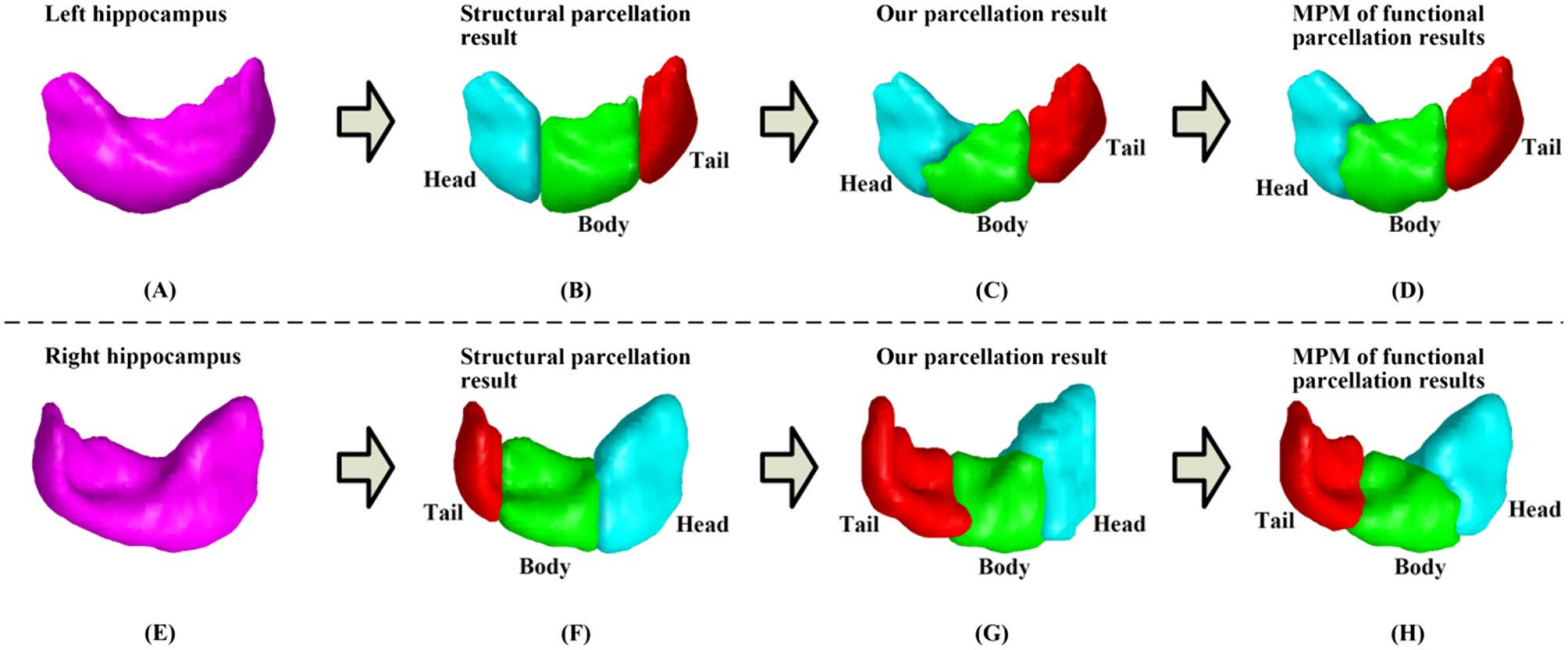
Hippocampus parcellation results with head, body, and tail parcels generated by structural parcellation method and proposed method. Up and down panels show left and right hippocampus parcellation results, respectively. (**A**, **E**) hippocampus segmentation results based on T1 image of one randomly selected subject from the 20 subjects from NewYork_b dataset, (**B**, **F**) structural hippocampus parcellation results of the randomly selected subject generated by structural parcellation method, (**C**, **G**) functional hippocampus parcellation results of the randomly selected subject generated by proposed method, (**D**, **H**) maximum probabilistic maps (MPM) of functional hippocampus parcellation results of the 20 subjects generated by proposed method. The figure was drawn by using BrainNet Viewer (BrainNet Viewer 1.7) (https://www.nitrc.org/projects/bnv/).

The hippocampus parcellation results with three parcels were obtained by using the proposed semi-supervised clustering based brain parcellation method. For each of the hippocampus parcels, a probability map was firstly obtained by calculating each voxel’s frequency belonging to head, body, or tail parcels across all of the 20 subjects from the NewYork_b dataset, respectively. Subsequently, based on the probability maps of hippocampus parcels, a group parcellation result, i.e., a maximum probabilistic map, was obtained by setting each voxel’s parcel label as the one that was associated with the maximal value among the probability values corresponding to the three hippocampus parcels. Figure 1 shows bilateral hippocampus segmentation results based on T1-weighted image of a randomly selected subject in panels (A and E), corresponding bilateral structural and functional parcellation results of the same subject generated by structural parcellation method and proposed method are shown in panels (B and F) and (C and G), respectively, and bilateral maximum probabilistic maps of functional parcellation results for all of the 20 subjects are shown in panels (D and H), respectively. It is noteworthy that head, body, and tail parcels of the maximum probabilistic maps occupied (left: 39.5%, 29.5%, and 31.0%) and (right: 42.1%, 28.7%, and 29.2%) of the whole hippocampus volume, while the respective values for the structural parcellation adopted as prior information were (left: 47.6%, 36.3%, and 16.1%) and (right: 49.1%, 35.3%, and 15.6%), indicating that the functional parcels of the hippocampus obtained by using the proposed method were not biased to the structural parcellation. We can see that the tail parcel obtained by the proposed method was larger than the one obtained by the structural parcellation method. The main reason might be that the boundary of the tail parcels has big differences between functional and structural neuroanatomy. These results demonstrated that the hippocampus was successfully parcellated into head, body, and tail parcels along the hippocampal longitudinal axis by the proposed method^2, 19^.

#### Validation through functional connectivity analysis

**Figure 2:**
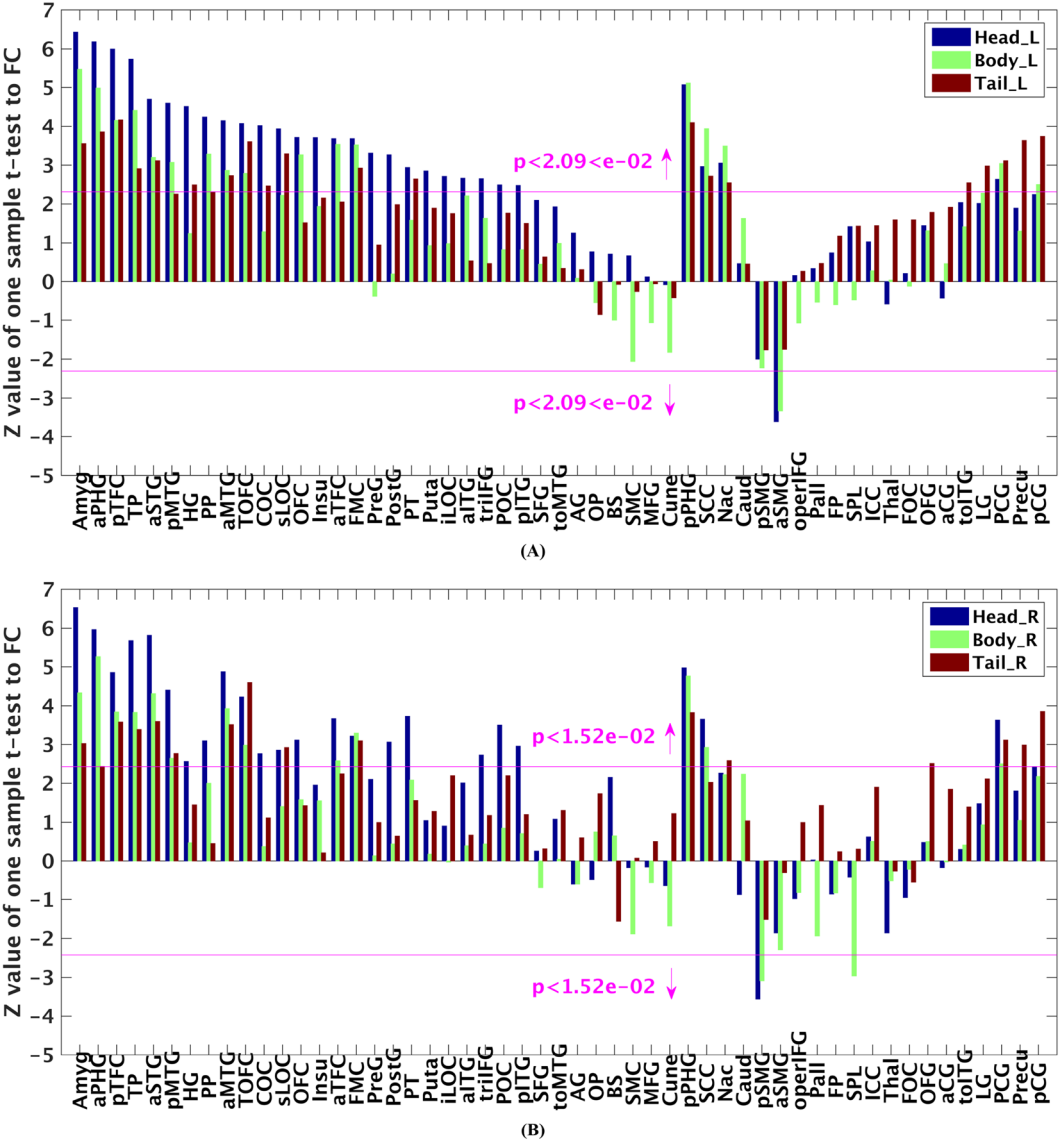
Whole-brain functional connectivity patterns of bilateral hippocampus parcels. (**A**, **B**) show *z* values of one sample t-test to functional connectivity (FC) between each hippocampus parcel and each ipsilateral brain region (obtained from Harvard–Oxford structural atlas) for left and right hemisphere, respectively. Particularly, in panels (**A**, **B**), the ordinate represents the *z* value obtained by applying one sample t-test to functional connectivity between brain regions across the 20 subjects from NewYork_b dataset, and the bars beyond the horizontal pink lines indicate that the false discovery rate (FDR) corrected *p* value of one sample t-test is smaller than 0.05. Abbreviations for brain regions obtained from Harvard–Oxford structural atlas: Amygdala, Amyg; Parahippocampal Gyrus, anterior division, aPHG; Temporal Fusiform Cortex, posterior division, pTFC; Temporal Pole, TP; Superior Temporal Gyrus, anterior division, aSTG; Middle Temporal Gyrus, posterior division, pMTG; Heschl’s Gyrus, HG; Planum Polare, PP; Middle Temporal Gyrus, anterior division, aMTG; Temporal Occipital Fusiform Cortex, TOFC; Central Opercular Cortex, COC; Lateral Occipital Cortex, superior division, sLOC; Frontal Orbital Cortex, OFC; Insular Cortex, Insu; Temporal Fusiform Cortex, anterior division, aTFC; Frontal Medial Cortex, FMC; Precentral Gyrus, PreG; Postcentral Gyrus, PostG; Planum Temporale, PT; Putamen, Puta; Lateral Occipital Cortex, inferior division, iLOC; Inferior Temporal Gyrus, anterior division, aITG; Inferior Frontal Gyrus, pars triangularis, triIFG; Parietal Operculum Cortex, POC; Inferior Temporal Gyrus, posterior division, pITG; Superior Frontal Gyrus, SFG; Middle Temporal Gyrus, temporooccipital part, toMTG; Angular Gyrus, AG; Occipital Pole, OP; Brain-Stem, BS; Juxtapositional Lobule Cortex (i.e., Supplementary Motor Cortex), SMC; Middle Frontal Gyrus, MFG; Cuneal Cortex, Cune; Parahippocampal Gyrus, posterior division, pPHG; Subcallosal Cortex, SCC; Accumbens, Nac; Caudate, Caud; Supramarginal Gyrus, posterior division, pSMG; Supramarginal Gyrus, anterior division, aSMG; Inferior Frontal Gyrus, pars opercularis, operIFG; Pallidum, Pall; Frontal Pole, FP; Superior Parietal Lobule, SPL; Intracalcarine Cortex, ICC; Thalamus, Thal; Frontal Operculum Cortex, FOC; Occipital Fusiform Gyrus, OFG; Cingulate Gyrus, anterior division, aCG; Inferior Temporal Gyrus, temporooccipital part, toITG; Lingual Gyrus, LG; Paracingulate Gyrus, PCG; Precuneous Cortex, Precu; Cingulate Gyrus, posterior division, pCG. Abbreviations for hippocampus parcels: left head, body, and tail parcels are denoted by Head_L, Body_L, and Tail_L, respectively; similarly, right head, body, and tail parcels are denoted by Head_R, Body_R, and Tail_R, respectively. The figure was drawn by using MATLAB (R2016b version 9.1.0.441655) (https://www.mathworks.com/).

As shown in Fig. 2, the head, body, and tail parcels had distinctive whole-brain functional connectivity patterns, which indicated that the three parcels of hippocampus might have different functions^37,53^. For both left and right three parcels shown in panels (A) and (B) of Fig. 2, the head parcel had significant functional connectivity with most number of the brain regions, while least number of brain regions significantly connected with the body parcel at a false discovery rate (FDR) threshold of *p* < 0.05. In particular, the head parcel shows preferential functional connectivity (i.e., strongest significant functional connectivity among the three parcels) with a large number of brain regions from temporal lobe (anterior division of superior temporal gyrus, anterior and posterior division of middle temporal gyrus, anterior and posterior division of inferior temporal gyrus, anterior and posterior division of temporal fusiform cortex, temporal pole, heschl’s gyrus, planum polare, and planum temporal), frontal lobe (frontal orbital cortex, pars triangularis of inferior frontal gyrus, central opercular cortex, and precentral gyrus), parietal lobe (parietal operculum cortex, and postcentral gyrus), occipital lobe (inferior division of lateral occipital cortex), limbic lobe (amygdala, and anterior division of parahippocampal gyrus). While the tail parcel shows preferential functional connectivity with several other brain regions, including lingual gyrus, precuneous, and posterior division of cingulate. In these findings, a part of the preferential functional connectivity between the head parcel and brain regions, including temporal pole, amygdala, and anterior parahippocampal gyrus, was reported in previous studies^54–56^, and the preferential functional connectivity between the tail parcel and brain regions, including lingual gyrus, precuneous, and posterior cingulate, was also reported in previous studies^54,56^. Therefore, the current findings enriched previous studies for the functional connectivity of hippocampus parcels^54–56^.

#### Validation through analyzing healthy adult aging of functional connectivity

**Figure 3:**
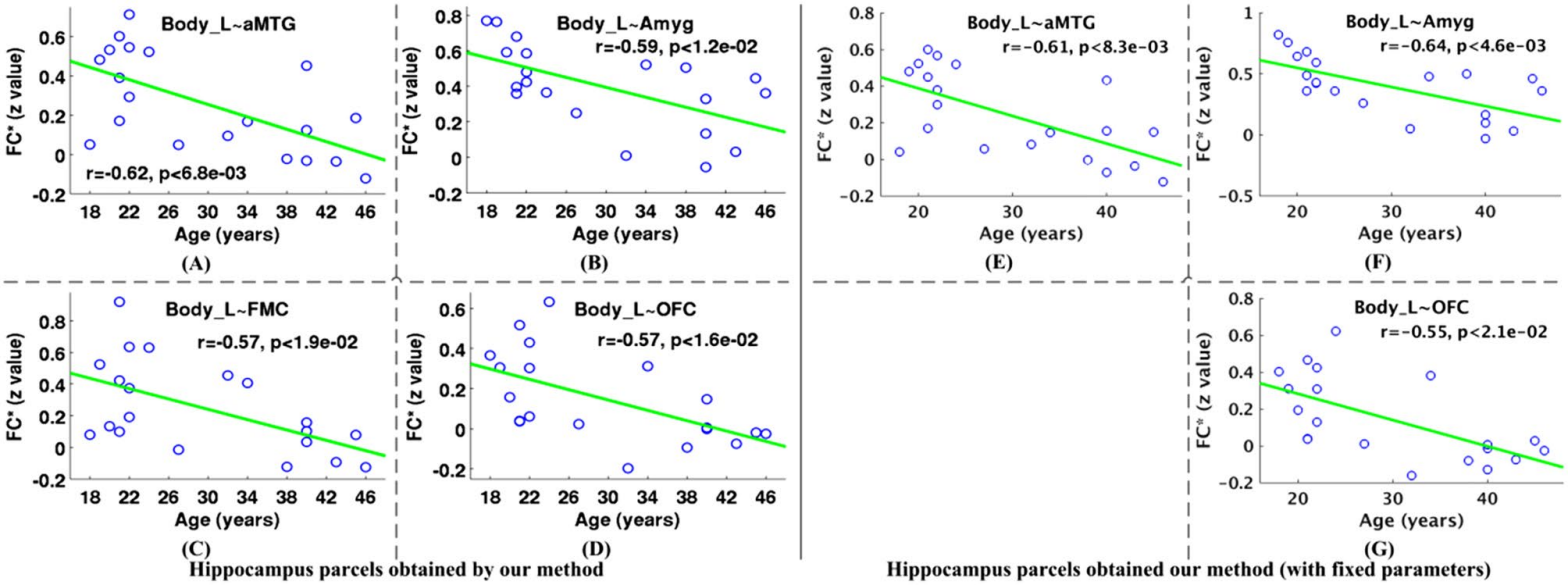
Effects of healthy adult aging on significant functional connectivity of hippocampus parcels based on resting state fMRI data of NewYork_b dataset. In panels (**A**–**G**), there are relationships that the hippocampus parcels’ functional connectivity significantly anticorrelated with age across 20 healthy adult subjects from the NewYork_b dataset (18–46 years old) (*p* < 0.05, FDR corrected), which indicates that increasing age is accompanied by decreased hippocampus parcels’ functional connectivity. Hippocampus parcels used for exploring aging effects of their functional connectivity were obtained by our method, and our method with a fixed parameter setting $\left( {\alpha = 1,\lambda = 1} \right)$. About related abbreviations, please refer to Fig. 2. The figure was drawn by using MATLAB (R2016b version 9.1.0.441655) (https://www.mathworks.com/).

We explored the effects of healthy adult aging on the significant functional connectivity of hippocampus parcels, and hippocampus parcels were obtained by using our proposed method and this method without parameter optimization, respectively. As shown in Fig. 3, the hippocampus parcels’ functional connectivity significantly anticorrelated with the age ranging from 18 to 46 years old for the 20 healthy adult subjects based on resting state fMRI data of NewYork_b dataset at a FDR threshold of *p* < 0.05. In particular, the left body parcel’s functional connectivity with four brain regions, including anterior division of middle temporal gyrus, amygdala, frontal medial cortex, and frontal orbital cortex, significantly anticorrelated with the age (Fig. 3A–G). These results demonstrated that increasing age was accompanied by decreased functional connectivity of the hippocampus parcel (i.e., left body parcel) in the adult aging span (18–46 years old).

**Figure 4:**
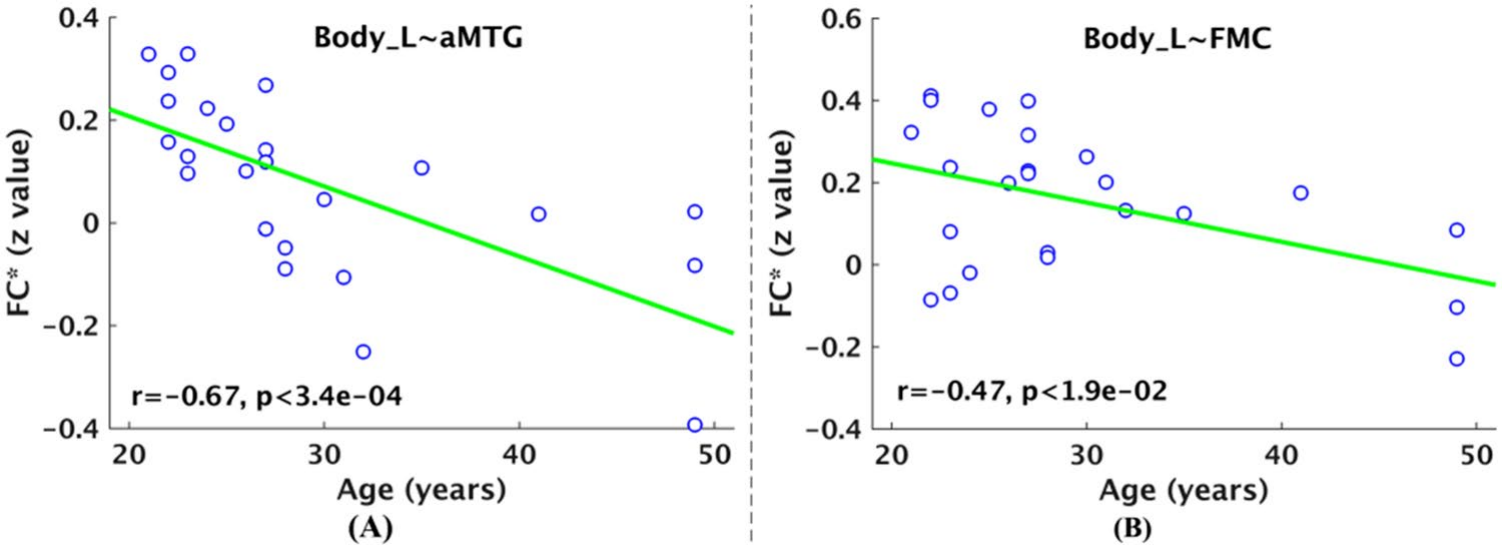
Effects of healthy adult aging on significant functional connectivity of hippocampus parcels based on resting state fMRI data of NewYork_Test-Retest_Reliability. In panels (**A**, **B**), there are two relationships that the hippocampus parcels’ functional connectivity significantly anticorrelated with age across 24 healthy adult subjects from the NewYork_Test-Retest_Reliability dataset (21–49 years old) (*p* < 0.05, FDR corrected), which indicates that the aging effects of hippocampus parcels’ functional connectivity were partially observed again in the second dataset. About related abbreviations, please refer to Fig. 2. The figure was drawn by using MATLAB (R2016b version 9.1.0.441655) (https://www.mathworks.com/).

In addition, the hippocampus parcels’ functional connectivity significantly anticorrelated with the age ranging from 21 to 49 years old for the 24 healthy adult subjects based on resting state fMRI data of NewYork_Test-Retest_Reliability at a FDR threshold of *p* < 0.05. Particularly, the left body parcel’s functional connectivity with the same two brain regions, i.e., anterior division of middle temporal gyrus, and frontal medial cortex, significantly anticorrelated with the age (Fig. 4A, B). The left body parcel’s functional connectivity with the other two brain regions, i.e., amygdala and frontal orbital cortex, exhibited a trend towards anticorrelation with the age. The correlation coefficients for amygdala and frontal orbital cortex were − 0.23 and − 0.12, respectively. These results demonstrated that the aging effects of hippocampus parcels’ functional connectivity were moderately reproducible.

### Comparison with three state-of-the-art brain parcellation methods on functional homogeneity

**Figure 5:**
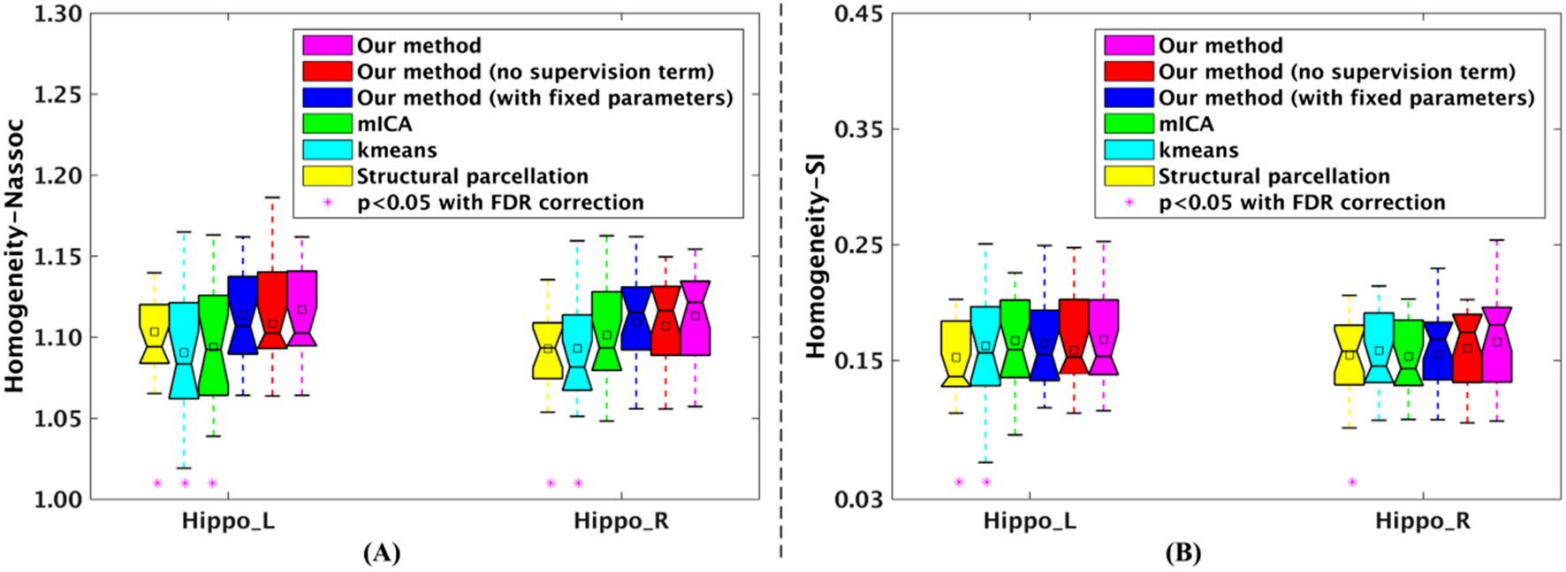
Comparisons of the functional homogeneity, measured by Nassoc and SI values, of bilateral hippocampus parcellation results of the 20 subjects from NewYork_b dataset generated by proposed method with the structural parcellation method, *k*-means clustering based brain parcellation method (kmeans), the masked independent component analysis based method (mICA), the proposed method without supervision term, and the proposed method with a fixed parameter setting $\left( {\alpha = 1,\lambda = 1} \right)$, respectively. The larger Nassoc or SI value indicates that the parcellation result is more functional homogeneous. Stars indicate the comparisons between proposed method and other three methods, the proposed method without supervision term, and the proposed method with the fixed parameter setting $\left( {\alpha = 1,\lambda = 1} \right)$ that are statistically significantly different, which were identified by two sample t-test at a threshold of *p* < 0.05 using false discovery rate (FDR) correction for multiple comparisons. On each box, the central mark is the median, and edges of the box are the 25th and 75th percentiles. Abbreviations: normalized association, Nassoc; silhouette width, SI; left hippocampus, Hippo_L; right hippocampus, Hippo_R. The figure was drawn by using MATLAB (R2016b version 9.1.0.441655) (https://www.mathworks.com/).

The effectiveness of the proposed method is validated through comparing with this method without parameter optimization. Figure 5 shows that the proposed method yielded more homogeneous parcellations for both left and right hippocampus after parameter optimization. These results demonstrated that proposed method with parameter optimization could generate brain parcellation results with higher functional homogeneity.

Additionally, we compared proposed method with the structural parcellation method, the masked ICA based brain parcellation method and the *k*-means clustering based brain parcellation method on functional homogeneity, which was measured by normalized association and modified silhouette width, respectively. As shown in Fig. 5, for both left and right hippocampus parcellations, the normalized association (Nassoc) and silhouette width (SI) values of parcellation results of the 20 subjects from NewYork_b dataset generated by our proposed method were larger than those generated by other three brain parcellation methods and the proposed method without supervision term, and the stars indicate that results of these comparisons from two sample t-tests are statistically significantly different (*p* < 0.05, FDR corrected for multiple comparisons). These results demonstrated that proposed method with supervision term could generate brain parcellation results with higher functional homogeneity.

### Test–retest reproducibility for brain parcellation

**Figure 6:**
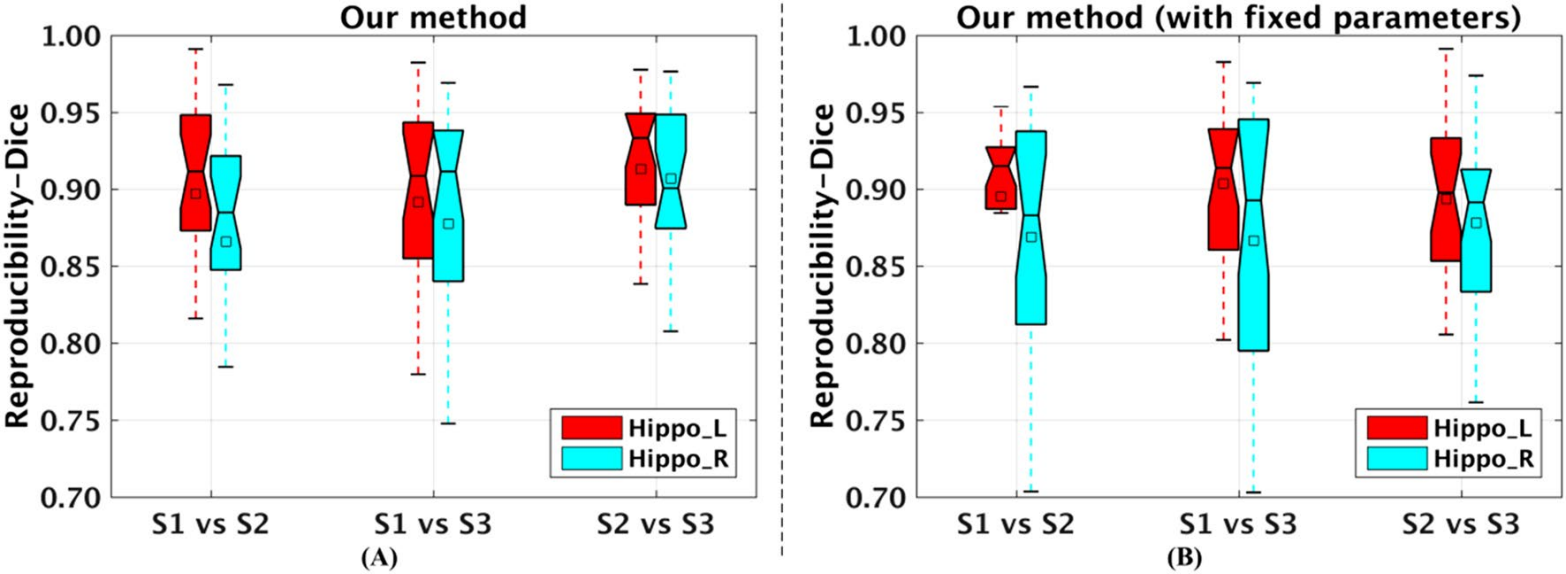
Test–retest reproducibility of the proposed method and this method without parameter optimization (i.e., with fixed parameters $\alpha = 1$ and $\lambda = 1$) for hippocampus parcellation between different sessions at subject level: box plots of Dice coefficients between parcellation results of the same subject from different sessions across 24 subjects from NewYork_Test-Retest_Reliability dataset at subject level. On each box, the central mark is the median, and edges of the box are the 25^th^ and 75^th^ percentiles. Abbreviations: left hippocampus, Hippo_L; right hippocampus, Hippo_R; Session 1, S1; Session 2, S2; Session 3, S3. The figure was drawn by using MATLAB (R2016b version 9.1.0.441655) (https://www.mathworks.com/).

**Figure 7:**
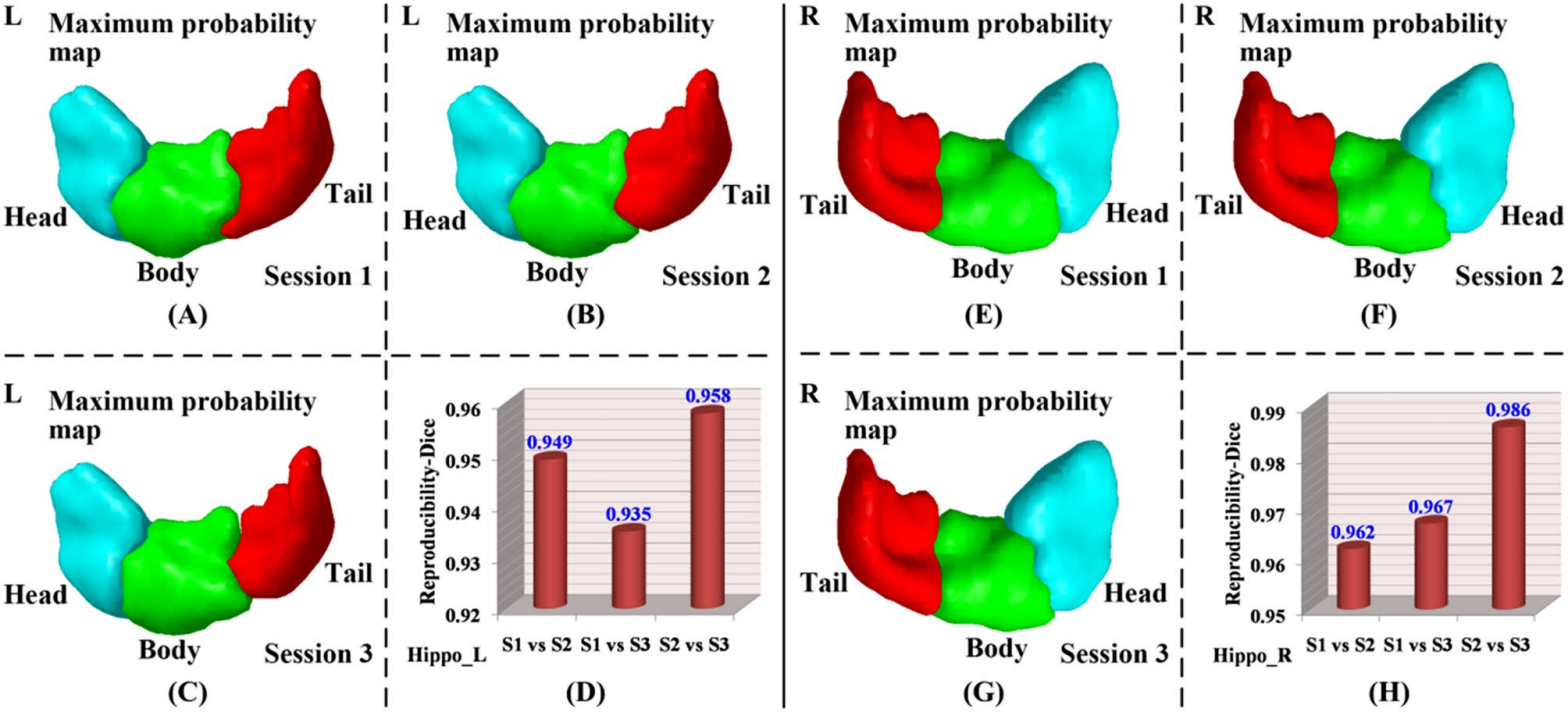
Test–retest reproducibility of the proposed method for hippocampus parcellation between different sessions at group level. (**A**–**C**, **E**–**G**) maximum probability maps of left and right hippocampus parcellation results at different sessions, respectively, (**D**, **H**) Dice coefficients between maximum probability maps of left and right hippocampus parcellation results from different sessions, respectively. Abbreviations: left, L; right, R. About other related abbreviations, please refer to Fig. 6. The (**A**–**C**, **E**–**G**) were drawn by using BrainNet Viewer (BrainNet Viewer 1.7) (https://www.nitrc.org/projects/bnv/), the (**D**, **H**) were drawn by using Microsoft Excel 2010 (https://www.microsoft.com/en-us/microsoft-365/previous-versions/office-2010).

As shown in Figs. 6A and 7, parcellation results generated by our proposed method were highly reproducible across different time points at both subject and group level. At subject level, the average Dice coefficients for 24 subjects’ hippocampus parcellation results of session1 (S1) versus S2, S1 versus S3, and S2 versus S3 were (left: 0.897, 0.892, and 0.913) and (right: 0.866, 0.878, and 0.907), respectively as shown in Fig. 6A, demonstrating that the proposed method had good test–retest reproducibility. We also evaluated the reproducibility of maximum probability maps between different sessions at group level. The Dice coefficients of maximum probability maps between different sessions were above 0.935 (S1 vs. S3 of left hippocampus) as shown in Fig. 7, further indicating that our proposed method had good test–retest reproducibility.

In addition, parcellation results generated by our proposed method without parameter optimization were also highly reproducible across different time points. At subject level, the average Dice coefficients for 24 subjects’ hippocampus parcellation results of S1 versus S2, S1 versus S3, and S2 versus S3 were (left: 0.895, 0.904, and 0.894) and (right: 0.869, 0.867, and 0.878), respectively as shown in Fig. 6B. At group level, the Dice coefficients for maximum probability maps of S1 versus S2, S1 versus S3, and S2 versus S3 were (left: 0.967, 0.963, and 0.957) and (right: 0.961, 0.956, and 0.955), respectively, which were above 0.955 (S2 vs. S3 of right hippocampus). These results demonstrated that the test–retest reproducibility was comparable between our proposed method and this method without parameter optimization at both subject and group level. However, brain parcellation results obtained by our proposed method with parameter optimization were more functionally homogeneous than this method with fixed parameter setting.

## Discussion

We proposed a novel semi-supervised clustering based brain parcellation method, which was validated through parcellating hippocampus, and the hippocampus was successfully parcellated into heady, body, and tail parcels along the hippocampal longitudinal axis (Fig. 1)^2,19^. The three parcels were applied to analyzing the distinctions and aging of their functional connectivity (Figs. 2 and 3, respectively), and the aging effects of hippocampus parcels’ functional connectivity were moderately reproducible (Fig. 4). Compared with the state-of-the-art brain parcellation methods, the proposed method had superior performance on functional homogeneity (Fig. 5). Furthermore, the proposed method had good test–retest reproducibility of the same brain’s parcellation results obtained from resting state fMRI data scanned at three different time points (Figs. 6 and 7).

We generalized the normalized cut algorithm, implemented by optimizing normalized association as objective function, to overcome the imaging noise for obtaining reliable brain parcellation by integrating prior and spatial information into the objective function defined in Eq. (3). The parameters $\alpha$ and $\lambda$ in Eq. (3) are weight factors for balancing among data term, supervision term, and spatial regularization term so the parcellation results generated by the proposed method depends on the two parameters. For obtaining more homogeneous parcellation results, the parameters were selected by a constrained bi-level programming optimization method defined in Eq. (8)^57,58^. The optimized parameter setting $\left( {\alpha_{*} ,\lambda_{*} } \right)$ guaranteed that the obtained parcellation results were spatially continuous (Fig. 1), and had optimal clustering quality (Fig. 5).

The proposed method was validated through parcellating the hippocampus into head, body, and tail parcels. The hippocampus parcels had distinctive preferential functional connectivity patterns (Fig. 2), which indicated that the three hippocampus parcels might be responsible for different functions^37,53^. The head parcel had preferential functional connectivity with brain regions distributing over the whole brain. Surprisingly, the head parcel preferentially connected with almost all brain regions of the temporal lobe except posterior division of superior temporal gyrus with a few voxels excluded in functional connectivity analysis. For example, the temporal pole had stably preferential functional connectivity with head parcel, which was consistent with findings in other studies^54,56^; a few brain regions of frontal lobe, such as frontal orbital cortex, had preferential functional connectivity with head parcel, which was supported by homologous anatomical connectivity found in the rodent studies^59^; there were part of brain regions of parietal lobe, occipital lobe, and limbic lobe, which preferentially functionally connected with head parcel. The head parcel’s preferential functional connectivity with amygdala and anterior parahippocampal gyrus, two brain regions of limbic lobe, was consistent with previous studies^55,56^. While the tail parcel had preferential functional connectivity with several other brain regions lingual gyrus, precuneous, and posterior cingulate, which was reported in previous studies as well^54,56^. These findings for the functional connectivity of hippocampus parcels enriched previous studies^54–56^, which might promote to explore the mechanism of hippocampus related aging and neuropsychiatric diseases, such as Alzheimer’s disease, anxiety disorder, depression, and schizophrenia^60^.

A great number of neuroimaging studies have investigated the effects of aging on hippocampus formation, which involved in age-related changes in functional connectivity, volume, metabolism, and stimulus-induced activation, etc.^14,60–65^. To our knowledge, this is first study to analyze changes in three hippocampus parcels’ functional connectivity based on resting state fMRI data. We found some significantly negative correlations between hippocampus parcel’ functional connectivity and age ranging from 18 to 46 years old across 20 healthy adult subjects based on resting state fMRI data of NewYork_b dataset (Fig. 3). The functional connectivity of hippocampus parcels, significantly negatively correlated with age, included connections between left body parcel and four brain regions, including anterior division of middle temporal gyrus, amygdala, frontal medial cortex, and frontal orbital cortex. In addition, the functional connectivity between left body parcel and two of these brain regions, i.e., anterior division of middle temporal gyrus, and frontal medial cortex, also significantly negatively correlated with age ranging from 21 to 49 years old across 24 healthy adult subjects based on resting state fMRI data of NewYork_Test-Retest_Reliability dataset (Fig. 4). These negative correlations demonstrated that increasing age was accompanied by decreased functional connectivity of the hippocampus parcel in the adult aging span, which might be associated with the hippocampus volume loss from about 20 years old reported in previous studies^66–68^. In this study, only observed left body parcel’ functional connectivity was significantly affected by aging. Simultaneously, we observed that there was a trend for the volume of the left body parcel to be anticorrelated with age (*r* = − 0.11). The reason for aging effects of left body parcel’ functional connectivity might be the greatest volume reduction of the left body parcel observed in a previous aging study^69^. Therefore, the hippocampus parcel’ functional connectivity might be an important biomarker in normal brain aging based on resting state fMRI data^14,62–65^.

Since there has been a large quantity of brain’s structural/functional knowledge, we adopted the prior knowledge as supervision information in a semi-supervised clustering method within a graph partitioning framework for obtaining reliable brain parcellation with improved functional homogeneity shown in Fig. 5^47^. There are two parameters $\alpha$ and $\lambda$ in the proposed semi-supervised clustering based brain parcellation method, which is needed to be tuned for achieving spatially and functionally consistent brain parcellation. Based on the parameter setting $({\alpha }_{*},{\lambda }_{*})$ selected by a constrained bi-level programming optimization method in the hippocampus parcellation as defined in Eq. (8)^57, 58^, the proposed method could generate more functionally homogeneous parcellation results than this method without parameter optimization (Fig. 5). Simultaneously, the proposed method could generate parcellation results with significantly higher functional homogeneity than the structural brain parcellation method^1–4^, masked ICA based brain parcellation method^13,15,70^, and *k*-means clustering based brain parcellation method^37,71^, respectively (Fig. 5). Furthermore, hippocampus parcels obtained by the proposed method might be more sensitive to effects of aging on their functional connectivity than this method without parameter optimization. The main reason is that additional aging effects on functional connectivity of left body parcel and frontal orbital cortex were found by the proposed method.

Through hippocampus parcellation, we have performed a test–retest study of the parcellation results based on 3 repeated resting state fMRI scans. The test–retest reproducibility experiments revealed that our proposed method could obtain reproducible parcellation results of the same brain based on resting state fMRI data collected at different time points, which was comparable with our proposed method without parameter optimization (Fig. 6). Nevertheless, our proposed method with parameter optimization could generate brain parcellation results with higher functional homogeneity than this method with fixed parameters (Fig. 5).

In conclusion, we proposed a novel semi-supervised clustering based brain parcellation method, whose parameter setting was tuned by a constrained bi-level programming optimization method. The results of validation experiments through hippocampus parcellation have demonstrated that the proposed method could generate three meaningful hippocampus parcels along its longitudinal axis with distinctive whole-brain functional connectivity patterns, and was applied to explore the effects of healthy adult aging on hippocampus parcels’ functional connectivity based on resting state fMRI data. In the validation through on hippocampus parcellation, the proposed method had better performance on functional homogeneity than the state-of-the-art brain parcellation methods, and had good test–retest reproducibility for parcellation results obtained from resting state fMRI data scanned at different time points. The proposed method will be further validated by parcellating other brain structures.

## Methods

### Resting state fMRI data and preprocessing

The resting state fMRI data of NewYork_b dataset, including 20 healthy subjects (8 males, 18–46 years old)^72^. The dataset is publicly available, which was obtained from http://fcon_1000.projects.nitrc.org. The scanning parameters of the fMRI data are repetition time [TR] = 2 s, voxel size = 3 × 3 × 4 mm^3^, in-plane matrix = 64 × 80, slices = 33, number of time point = 175. The scanning parameters of the 20 subjects’ sagittal T1-weighted images are voxel size = 1.33 × 1 × 1 mm^3^, in-plane matrix = 256 × 256, sagittal slices = 128.

The extra resting state fMRI dataset of NewYork_Test-Retest_Reliability dataset with 25 healthy subjects (10 males, 21–49 years old), each of them having 3 repeated scans, was used to evaluate our method’s test–retest reproducibility^73^. The extra dataset is publicly available, which was obtained from http://fcon_1000.projects.nitrc.org. For each subject, imaging data of time points 2 and 3 was collected 5–16 months (mean 11 ± 4) after time point 1 with interval of about 30 min. The data collected at time points 1, 2, and 3 are referred to as session 1, session 2, and session 3, respectively. The scanning parameters of the fMRI data are repetition time [TR] = 2 s, voxel size = 3 × 3 × 3 mm^3^, in-plane matrix = 64 × 64, slices = 39, number of time point = 197. The scanning parameters of the 25 subjects’ sagittal T1-weighted images are voxel size = 1 × 1 × 1 mm^3^, in-plane matrix = 256 × 256, sagittal slices = 176.

The study was performed in accordance with the Health Insurance Portability and Accountability Act (HIPAA) guidelines (http://fcon_1000.projects.nitrc.org/fcpClassic/FcpTable.html), which was approved by the New York University. All subjects participating in this study provided written informed consent.

The resting state fMRI data was preprocessed based on our preliminary study with following steps^45^: (1) head motion correction, (2) scaling the intensity of each fMRI scan after motion correction to yield a whole-brain mean value of 10,000, (3) temporally band-pass filtering (0.01–0.08 Hz), (4) regressing out of a set of nuisance signals including mean of white matter signals, mean of cerebrospinal fluid signals, and six motion parameters, (5) nonlinearly normalizing into Montreal Neurological Institute (MNI) space with resolution 3 × 3 × 3 mm^3^, (6) spatially smoothing with a 6 mm full width at half maximum (FWHM) Gaussian kernel. The nonlinear normalization of fMRI data was implemented using DARTEL of SPM12 with the deformation fields of their co-registered T1-weighted images. One subject from the NewYork_Test-Retest_Reliability dataset was removed due to failed image preprocessing.

### Semi-supervised clustering for graph partition

The graph theory based semi-supervised clustering algorithm firstly constructs an undirected graph $G = \left( {V,E} \right)$, which models data points to be clustered as graph nodes $V$ and connections between graph nodes as edges $E$^47,52^. Each graph edge is associated with a weight value $a_{uv}$ measuring the similarity of nodes $u$ and $v$. Then, the graph is partitioned into subgraphs $G_{c} = \left( {V_{c} ,E_{c} } \right),c = 1, \ldots ,k$ guided by prior knowledge, and $k$ is pre-specified number of cluster. Consequently, the graph nodes $V$ is partitioned into $k$ clusters $V_{1}$, …, $V_{k}$.

#### Semi-supervised graph partition

In the semi-supervised graph partition, the graph is partitioned into subgraphs by (1) maximizing similarity of nodes within each subgraph, (2) encouraging in accordance to prior knowledge, such as partially manually labeled data points, and (3) enhancing spatial connectedness of partitioned clusters, which was preliminarily described in our previous study^45^. In particular, the overall similarity of nodes within ${\varvec{k}}$ subgraphs is measured by the data term, namely normalized association ${\varvec{Nassoc}}_{{\varvec{k}}}$^47,52,74,75^, which has been detailedly described in our preliminary study^45^.

The similarity between the partitioned clusters and the prior information is measured by the total reward gains of pairs of nodes within each subgraph, computed by supervision term as^47^

1 $$S_{k} = \mathop \sum \limits_{c = 1}^{k} \frac{{\mathop \sum \nolimits_{i = 1}^{k} \mathop \sum \nolimits_{{u,v \in P_{i} \cap V_{c} }} s_{uv} }}{{{\text{degree}}\left( {V_{c} } \right)}} + \mathop \sum \limits_{c = 1}^{k} \frac{{\mathop \sum \nolimits_{i = 1}^{k - 1} \mathop \sum \nolimits_{j = i + 1}^{k} \mathop \sum \nolimits_{{u \in P_{i} \cap V_{c} ,v \in P_{j} \cap V_{c} }} s_{uv} }}{{{\text{degree}}\left( {V_{c} } \right)}}$$

where $P_{i} , \,i = 1, \ldots ,k$ are labeled data points of each cluster provided by the prior information, $s_{uv}$ equals 1 if $u,v \in P_{i} ,i = 1, \ldots ,k$, $s_{uv}$ equals -1 if $u \in P_{i} ,v \in P_{j} ,i \ne j,i,j = 1, \ldots ,k$, and 0 otherwise*.*

The spatial connectedness of the partitioned clusters is enhanced by rewarding neighboring data points belonging to the same cluster, calculated by spatial regularization term as^49–51,76^

2 $$R_{k} = \mathop \sum \limits_{c = 1}^{k} \frac{{\mathop \sum \nolimits_{{u,v \in V_{c} }} e_{uv} }}{{\text{degree}\left( {V_{c} } \right)}},$$

where $e_{uv}$ equals 1 if graph nodes $u$ and $v$ are spatially nearest neighbors, and 0 otherwise.

Finally, the semi-supervised graph partition is solved by optimizing the following objective function as^47^

3 $$J\left( {\left\{ {V_{c} } \right\}_{c = 1}^{k} } \right) = {\text{argmax}}_{{V_{1} , \ldots ,V_{k} }} \left( {Nassoc_{k} + \alpha S_{k} + \lambda R_{k} } \right),$$

where $\alpha$ and $\lambda$ are weighting factors among data term, supervision term and spatial regularization term.

#### Weighted kernel *k*-means algorithm for graph partition

The optimization problem of graph partition modeled by Eq. (3) can be solved by using an iterative weighted kernel *k*-means algorithm^47,52^. At each iterative step ${\varvec{t}}$, the pseudo-distance from each node ${\varvec{u}}$ to every cluster ${\varvec{V}}_{{\varvec{c}}}^{{\left( {\varvec{t}} \right)}}$ is computed as

4 $$d\left( {u,V_{c}^{\left( t \right)} { }} \right) = k_{uu} - \frac{{2\mathop \sum \nolimits_{{v \in V_{c}^{\left( t \right)} }} w_{v} k_{uv} }}{{\mathop \sum \nolimits_{{v \in V_{c}^{\left( t \right)} }} w_{v} }},c = 1, \ldots ,k,$$

where $k_{uv}$ is the element of the kernel matrix $K$ for nodes $u$ and $v$, $K = D^{ - 1} \left( {A + \alpha S + \lambda R} \right)D^{ - 1}$, $A$ is the adjacency matrix with elements $a_{uv}$; $S$ is a matrix with elements $s_{uv}$; $R$ is a matrix with elements $e_{uv}$; $D$ is a diagonal matrix with elements $d_{uu} = \sum\nolimits_{v} {a_{uv} }$; $D^{ - 1}$ is the inverse of the matrix $D$; and $w_{v} = w_{vv}$ is the diagonal elements of the weight matrix $W = D$. Based on the distance measures, we can assign each graph node to a cluster label with the shortest pseudo-distance. The algorithm is described in Algorithm 1.
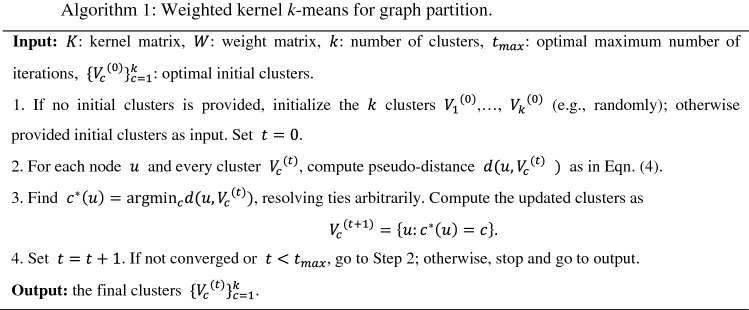


### Semi-supervised clustering for brain parcellation

For brain parcellation based on resting state fMRI data, it is assumed that voxels, belonging to the same functional unit, have functional signals that are highly correlated^23,24,31,77^, have functional signals that are short in functional distance^23^, or have similar functional connectivity patterns^37^. Based on the similarity of voxels’ functional signals or functional connectivity patterns, the semi-supervised clustering based brain parcellation typically consists of two components according to our preliminary studies^44–46^. Firstly, a similarity measure of functional signals for voxels within target region considered for parcellation is defined for the data term of normalized association^45^; partially labeled voxels derived from prior knowledge is adopted as supervision information for the supervision term defined in Eq. (1); the regularization term defined in Eq. (2), considering the spatial relationship between voxels, is adopted for spatially consistent brain parcellation. Secondly, a semi-supervised clustering algorithm is chosen to group the voxels into clusters. In the current study, the graph theory based semi-supervised clustering defined in Eq. (3) is applied to brain parcellation based on resting state fMRI data. The semi-supervised clustering based method is a general framework for brain parcellation, and the unsupervised brain parcellation method using normalized cut is a special case with weight factors $\alpha ,\lambda = 0$ in Eq. (3)^22,23,25^. For improving performance of brain parcellation on functional homogeneity, we adopt a constrained bi-level programming optimization method to select optimal weight factors $\alpha$ and $\lambda$ building on our preliminary studies^45,46^.

#### Similarity measure of functional signals for brain parcellation

The functional connectivity between functional signals is typically measured by their Pearson correlation coefficient^78^

5 $$r_{uv} = \frac{{\left( {1/T} \right)\mathop \sum \nolimits_{t = 1}^{T} \left( {I\left( {u,t} \right) - \overline{I}\left( u \right)} \right)\left( {I\left( {v,t} \right) - \overline{I}\left( v \right)} \right)}}{{SD_{I} \left( u \right)SD_{I} \left( v \right)}},$$

where column vectors $\overrightarrow {I\left( u \right)} = [I\left( {u,1} \right)$;*…*;$I\left( {u,T} \right)]$ and $\overrightarrow {I\left( v \right)} = [I\left( {v,1} \right)$;…;$I\left( {v,T} \right)]$ are normalized functional signals at voxels $u$ and $v$ with mean $\overline{I}\left( \cdot \right) = 1/T\sum\nolimits_{t = 1}^{T} I \left( { \cdot ,t} \right) = 0$ and standard deviation $SD_{I} \left( \cdot \right) = \sqrt {\left( {1/T} \right)\sum\nolimits_{t = 1}^{T} {\left( {I\left( { \cdot ,t} \right) - \overline{I}\left( \cdot \right)} \right)^{2} } } = 1$, and $T$ is the number of time points. Then, according to our preliminary studies the similarity measure of functional signals can be defined as^45,46^

6 $$a_{uv} = r_{uv} + 1,$$

where $r_{uv}$ is the Pearson correlation coefficient of functional signals between voxels $u$ and $v$ defined in Eq. (5).

#### Optimization of parameters for brain parcellation

To obtain a brain parcellation with spatially and functionally consistent clusters, it is desired that voxels within each cluster of a brain parcellation are spatially connected. The connectedness can be guaranteed by satisfying the topological property of the cluster using geodesic star convexity shape^79^. The geodesic shape enforces a voxel in each cluster that is connected to all other voxels of the cluster by at least one geodesic path entirely included in the cluster, i.e., any pair of voxels from the cluster can be linked by a geodesic path that is entirely within the cluster. For a parcellation of ${\varvec{V}}$ with ${\varvec{k}}$ clusters ${\varvec{V}}_{1}$,…,${\varvec{V}}_{{\varvec{k}}}$, we characterize the topological property of each cluster by geodesic star convexity. If the topological property of a cluster ${\varvec{V}}_{{\varvec{c}}}$ satisfies geodesic star convexity, we denote the connectedness of the cluster with ${\varvec{GSC}}_{{\varvec{c}}} = 1$, and otherwise 0. Therefore, for each cluster of a brain parcelllation without any spatially disconnected voxels, satisfying ${\varvec{GSC}}_{{\varvec{c}}} = 1 \;{\mathbf{for}}\;{\varvec{c}} = 1, \ldots ,{\varvec{k}}$.

Similar to most image segmentation tasks^49,50,76^, for brain parcellation results with the same quality, the smoother one is usually preferred. Given a brain parcellation of $V$ with $k$ clusters $V_{1}$,…, $V_{k}$, the smoothness can be measured based on its corresponding edge energy^49,50,76^

7 $$Sm = \frac{{N - \mathop \sum \nolimits_{u \in V} \mathop \sum \nolimits_{{v \in {\mathcal{N}}_{u} }} \chi \left( {u,v} \right)}}{N},$$

where $u,v \in V$ are two voxels within $V$, $N$ is the number of voxels within $V$, ${\mathcal{N}}_{u}$ is the spatially nearest neighboring voxels (e.g., 26-connected neighborhood) of $u$, and $\chi \left( {u,v} \right)$ is equal to 0 if the voxels $v$ and $u$ are in the same cluster, and 1 otherwise. Theoretically, the smoothness measure is inversely proportional to the length of the cluster boundaries^49,50,76^, i.e., higher smoothness is associated with shorter boundary length and vice versa.

Given a parameter setting $\left( {\alpha , \lambda } \right)$ in Eq. (3), a brain parcellation result can be obtained by optimizing the objective function of Eq. (3) by using the weighted kernel *k*-means algorithm (Algorithm 1) based on the similarity measure of functional signals defined in Eq. (6). The supervision and spatial regularization terms in the objective function are adopted for obtaining reliable brain parcellation. To achieve a more spatially consistent and functionally homogeneous brain parcellation, we adopt a bi-level programming optimization method for tuning the parameter setting $\left( {\alpha , \lambda } \right)$ in the objective function defined in Eq. (3). In particular, we firstly identify the parameter settings, defined by $p = \left( {\alpha , \lambda } \right) \in P$, that are able to generate spatially continuous parcellation results satisfying topological property of geodesic star convexity. Then, within the constrained parameter space we find the parameter $p_{*} = \left( {\alpha_{*} , \lambda_{*} } \right)$ that yields the parcellation result with maximal Nassoc value and optimal smoothness by sequentially optimizing the two objective functions, i.e., normalized association and smoothness, respectively. Mathematically, for parcellating $V$ into clusters $V_{1}$,…,$V_{k}$ by optimizing objective function of Eq. (3) with similarity measure defined in Eq. (6), the constrained bi-level programming optimization problem for tuning the parameter setting $\left( {\alpha , \lambda } \right)$ is defined as^57, 58^

8 $$\begin{aligned} & {\max}_{\alpha , \lambda } Sm\left( {\alpha ,\lambda } \right) = \frac{{N - \mathop \sum \nolimits_{u \in V} \mathop \sum \nolimits_{{v \in {\mathcal{N}}_{u} }} \chi \left( {u,v} \right)}}{N} \\ {\text{s}}.{\text{t}}.{ } & \left\{ {\begin{array}{*{20}l} {{\max}_{\alpha ,\lambda } Nassoc_{k} \left( {\alpha ,\lambda } \right) = \mathop \sum \limits_{c = 1}^{k} \frac{{{\text{links}}\left( {V_{c} ,V_{c} } \right)}}{{{\text{degree}}\left( {V_{c} } \right)}}} \hfill \\ {{\text{s}}.{\text{t}}.GSC_{{\text{c}}} \left( {\alpha ,\lambda } \right) = 1, \quad for\; c = 1, \ldots k} \hfill \\ \end{array} } \right. \\ \end{aligned}$$

where $\left( {\alpha , \lambda } \right)$ are the parameter setting in $P = \left\{ {\left( {\alpha , \lambda } \right) \in {\mathbb{R}}_{ \ge 0} \times {\mathbb{R}}_{ \ge 0} :{ }\alpha 1 \le \alpha \le \alpha 2, \lambda 1 \le \lambda \le \lambda 2} \right\}$, $\alpha 1, \alpha 2, \lambda 1, \lambda 2 \in {\mathbb{R}}_{ \ge 0}$, $Nassoc_{k}$ is the parcellation’s Nassoc value^45^, $Sm$ is the parcellation’s smoothness defined in Eq. (7), and $GSC_{c}$ measures each cluster’s topological property of connectedness. The algorithm is described in Algorithm 2.
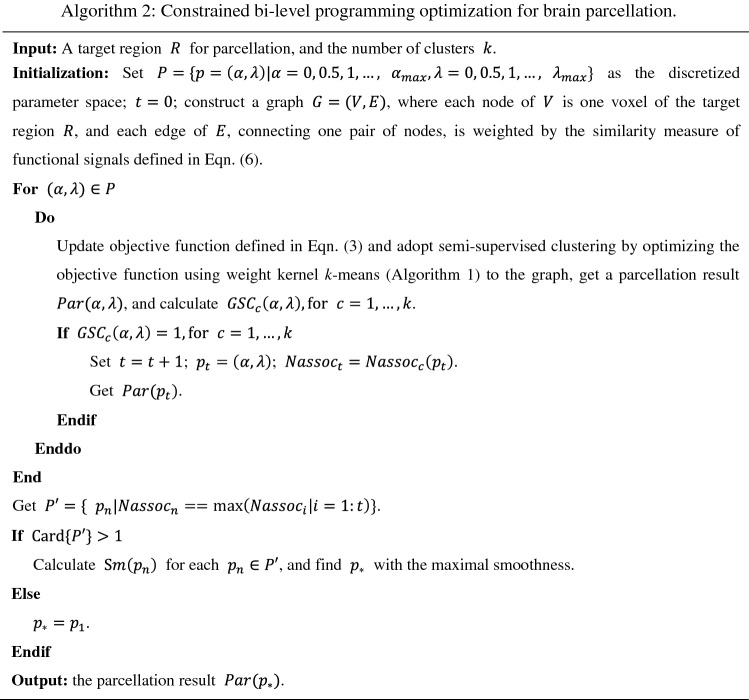


### Comparison with the state-of-the-art brain parcellation methods

We compared the proposed semi-supervised method with the state-of-the-art brain parcellation method, i.e., masked ICA based brain parcellation method, which has been successfully adopted for parcellating hippocampus at group level^13,15^. In the current study, the masked ICA based method was adopted to parcellate the brain structure at the individual subject level for comparison^70^. Particularly, the group independent components (ICs) are firstly estimated by using masked ICA, i.e., spatially restricted ICA on the brain structure considered for parcellation, based on resting state fMRI data in a group of subjects. Then, subject-specific ICs are estimated by using the dual regression, which are associated with the group ICs. Finally, for each subject, the brain structure’s parcelation results are obtained by computing the winner-take-all map of the estimated subject-specific ICs. In our experiments, we applied the masked ICA based method to parcellate the hippocampus based on the estimated subject-specific ICs.

Additionally, we compared the proposed semi-supervised method with *k*-means clustering based unsupervised method, which has been widely adopted in brain parcellation^37,71^. Particularly, elements $a_{uv}$ in the adjacency matrix $A$ (i.e., functional similarity matrix) are defined in Eq. (6). Then, the *k*-means clustering algorithm is applied to the rows of the adjacency matrix $A$, and assigning each voxel within the hippocampus to one of the *k* different clusters by minimizing the Euclidean distance between the voxel and the cluster centroid, and *k* is the clustering number. The labeling procedure is iteratively reassigning the voxel within the hippocampus until the intra-cluster distance across all of the *k* clusters reaches a minimum.

### Validation experiments

We validated the proposed method through hippocampus parcellation using resting state fMRI data of 20 subjects from NewYork_b dataset. In particular, we validated parcellation results through functional connectivity analysis and analyzing healthy adult aging of hippocampus parcels’ functional connectivity. Then, we compared the proposed method with the state-of-the-art brain parcellation methods with respect to their brain parcellation results’ functional homogeneity.

We finally evaluated the test–retest reproducibility of proposed method based on resting state fMRI data of 24 subjects from NewYork_Test-Retest_Reliability dataset, each of them having 3 repeated scans.

### Validation through hippocampus parcellation

#### Application of the proposed method to hippocampus parcellation

For validating the proposed method, the hippocampus was chosen as target region for parcellation. For each subject, bilateral hippocampus was segmented from the subject’s T1-weighted image using multi-atlas based local label learning method^80^. The existing neuroanatomy knowledge has suggested that the hippocampus could be parcellated into head, body, and tail parts based on sMRI^1–5^, referred to as structural parcellation method. Therefore, the hippocampus was parcellated into 3 clusters according to the prior knowledge based on resting state fMRI data in the current study.

For parcellating the hippocampus with the proposed semi-supervised method, the prior information, adopted as supervision information, was obtained from the subject-specific structural parcellation of the hippocampus for each subject. Instead of directly using the structural parcellation as the prior information, we identified a small region with homogeneous functional signals within each structural parcellation subregion to avoid any possible bias to the structural parcellation that might be inconsistent with the functional information. In particular, the structural parcellation method divided the hippocampus into three subregions, i.e., head, body and tail parcels, by two planes, determined by two landmarks uncal apex and the wing of the ambient cistern, that are normal to *y* axis in MNI space for each subject^1–5^. Then, the three structural subregions of the hippocampus were segmented into small functionally homogeneous regions according to each voxel’s functional consistency with its neighboring voxels using watershed segmentation algorithm, respectively^21^. The functional consistency at a voxel was calculated as the root mean square error to normalized functional signals between the voxel and voxels from its 26-connected neighborhood^31^. Finally, the most homogeneous three regions $P_{1} ,P_{2} ,P_{3}$, one from each structural subregion, with the smallest min-maxcut (Mcut) value, as defined by Eq. (9), were selected as prior information for functional parcellation of the hippocampus. The Mcut value is computed based on similarity measure defined in Eq. (6) as the inter-region to intra-region similarity ratio by^81^

9 $$Mcut_{k} = \mathop \sum \limits_{i = 1}^{k} \frac{{\mathop \sum \nolimits_{j = 1,j \ne i}^{k} {\text{links}}\left( {P_{i} ,P_{j} } \right)}}{{{\text{links}}\left( {P_{i} ,P_{i} } \right)}},$$

where $k = 3$ is the number of regions, and $Mcut_{k}$ measures the regions’ functional homogeneity.

Based on partially labeled voxels $P_{c} ,c = 1, \ldots ,3$ and similarity measure defined in Eq. (6), we applied the constrained bi-level programming optimization algorithm (Algorithm 2) to parcellate the hippocampus into three parcels.

#### Validation through functional connectivity analysis

We validated the proposed method through functional connectivity analysis with an assumption that different functional subregions should have distinctive functional connectivity patterns^37,53^. Firstly, the brain regions were obtained from the Harvard–Oxford structural atlas of the whole brain (distributed with the FSL software package at https://fsl.fmrib.ox.ac.uk/fsl/). The atlas includes forty eight cortical and eight subcortical brain regions in each hemisphere. In our experiments, besides the subcortical hippocampus, two cortical brain regions, i.e., posterior division of superior temporal gyrus and supracalcarine cortex, have a few voxels, which were excluded in the functional connectivity analysis. Secondly, for each hemisphere and every subject, the functional connectivity, measured by Pearson correlation coefficient ${\varvec{r}}_{{{\varvec{HO}}}}$, was calculated according to Eq. (5) between mean normalized functional signals within each parcellated parcel ${\varvec{H}}$ of the hippocampus and each brain region ${\varvec{O}}$ of the whole brain. Then, the Pearson correlation coefficient was converted into *z* value using Fisher’s transform to improve the normality computed by ${\varvec{z}}_{{{\varvec{HO}}}} = \frac{1}{2}{\mathbf{log}}\frac{{1 + {\varvec{r}}_{{{\varvec{HO}}}} }}{{1 - {\varvec{r}}_{{{\varvec{HO}}}} }}$^82^. Next, one sample t-test was applied to the normalized functional connectivity measures (*z* values) between each hippocampus parcel and each brain region across all subjects from NewYork_b dataset, and the *t* value of the statistics was transformed into *z* value. Finally, the hippocampus parcels’ functional connectivity with statistical significant was determined at a FDR threshold of *p* < 0.05.

#### Validation through analyzing healthy adult aging of functional connectivity

We validated the proposed method through analyzing the effects of aging on the hippocampus parcels’ functional connectivity because previous studies have implicated the hippocampus structure in normal brain aging^60,61^. In our experiments, we adopted linear model to explore the changes of functional connectivity based on resting state fMRI data from NewYork_b dataset in part of the adult age span (18–46 years old)^83^. In particular, for each pair of the hippocampus parcel ${\varvec{H}}$ and the brain region ${\varvec{O}}$ (from the Harvard–Oxford atlas) localized in the same hemisphere with significant functional connectivity, we applied age as the independent variable to predict their functional connectivity along with sex as a covariate modeled by a general multiple linear regression problem as follows^84–87^

10 $$\overrightarrow {FC} = \beta_{0} 1 + \beta_{1} \overrightarrow {Age} + \beta_{2} \overrightarrow {Sex} + {\mathbf{\mathcal{E}}},$$

where $\overrightarrow {FC} = [z_{HO} \left( 1 \right)$;…;$z_{HO} \left( M \right)]$ is a column vector with elements normalized functional connectivity measures of $M$ healthy adult subjects, and $z_{HO} \left( s \right), s = 1,..,M$ is the normalized functional connectivity between brain regions $H$ and $O$ for subject $s$. Similarly, $\overrightarrow {Age}$ and $\overrightarrow {Sex}$ are two column vectors with elements age and sex (female 0, and male 1) of the $M$ subjects, respectively, and $1$ and ${\mathbf{\mathcal{E}}}$ are two column vectors with $M$ elements all 1 and estimated noises, respectively. The parameters $\beta_{0}$, $\beta_{1}$ and $\beta_{2}$ were estimated regression coefficients^84–87^. Then, the functional connectivity was adjusted for the covariate sex by $\overrightarrow {FC} * = \overrightarrow {FC} - \beta_{2} \overrightarrow {Sex} - {\mathbf{\mathcal{E}}}$. Finally, the significant linear relationship between $\overrightarrow {FC} *$ and $\overrightarrow {Age}$ was obtained at a FDR threshold of *p* < 0.05.

We further validated the proposed method through analyzing the aging effects of the hippocampus parcels’ functional connectivity based on resting state fMRI data collected at three time points from NewYork_Test-Retest_Reliability dataset. The ages for subjects from NewYork_Test-Retest_Reliability dataset range from 21 to 49 years old, which are in almost the same adult age span as subjects’ age span from NewYork_b dataset. The hippocampus parcels’ functional connectivity measures were averaged across the three time points for analyzing the aging effects. Subsequently, the aging effects of the hippocampus parcels’ functional connectivity were analyzed similarly as described in Eq. (10). Finally, we obtained the significant linear relationships between $\overrightarrow {FC} *$ and $\overrightarrow {Age}$ at a FDR threshold of *p* < 0.05.

### Comparison with the state-of-the-art brain parcellation methods on functional homogeneity

We compared proposed method with this method without parameter optimization, i.e., the proposed method with a fixed parameter setting $\left( {\alpha = 1,\lambda = 1} \right)$. We also compared proposed method with the structural parcellation method, the masked ICA based brain parcellation method and *k*-means clustering based brain parcellation method on functional homogeneity. The structural parcellation method has been described in "Comparison with the state-of-the-art brain parcellation methods", which divided the hippocampus into head, body, and tail parcels using structural landmarks based on sMRI^1–4^. The other two methods for comparison are the masked ICA based brain parcellation method^13,15,70^ and *k*-means clustering based brain parcellation method^37,71^.

We adopted the normalized association and silhouette width (SI) to measure the functional homogeneity of hippocampus results, respectively. The normalized association, denoted by Nassoc, is detailedly defined in our preliminary study^45^, and a parcellation’s larger Nassoc value indicates its higher functional homogeneity. As to SI, the modified SI, adopted to measure the functional homogeneity of parcellation results with $k$ parcels $V_{c} ,c = 1, \ldots ,k$ of a given brain structure $V$ based on the resting state fMRI data, is defined as^22^

11 $$SI_{k} = \frac{1}{k}\mathop \sum \limits_{c = 1}^{k} \frac{{a_{c} - b_{c} }}{{{\max}\left\{ {a_{c} ,b_{c} } \right\}}},$$

where $a_{c} = \frac{1}{{n_{c} \left( {n_{c} - 1} \right)}}\sum\nolimits_{{u,v \in V_{c} ,u \ne v}} {a_{uv} }$, $b_{c} = \frac{1}{{n_{c} \left( {N - n_{c} } \right)}}\sum\nolimits_{{u \in V_{c} ,v \in \left( {V - V_{c} } \right)}} {a_{uv} }$, $n_{c}$ is the number of voxels within parcel $V_{c}$, $N$ is the number of voxels within the given brain structure $V$, and $a_{uv}$ is the similarity measure of functional signals defined in Eq. (6). A large value of $SI_{k}$ typically indicates a parcellation with highly homogeneous parcels.

### Test–retest reproducibility for brain parcellation

The reproducibility of parcellation results obtained from resting state fMRI data of different time points was evaluated at both subject and group level. In particular, at subject level the reproducibility of parcellation results of the same subject between different time points is measured by Dice coefficient^88^

12 $$Dice = \frac{1}{k}\mathop \sum \limits_{i = 1}^{k} \frac{{2\left| {X_{i} \bigcap {Y_{i} } } \right|}}{{\left| {X_{i} } \right| + \left| {Y_{i} } \right|}},$$

where $k$ is number of clusters, $X_{i}$ and $Y_{i} , i = 1, \ldots ,k$ are voxels of the $i$^th^ cluster in the two different parcellation results $\{ X_{i} \}_{i = 1}^{k}$ and $\left\{ {Y_{i} } \right\}_{i = 1}^{k}$, $X_{i} \bigcap {Y_{i} }$ is their intersection, and $\left| \cdot \right|$ is the cardinality of a set. The Dice’s coefficient is always in [0, 1]. Simultaneously, maximum probability maps are firstly obtained based on parcellation results of different time points. Then, the reproducibility of maximum probability maps between different time points is also measured by Dice coefficient defined by Eq. (12) at group level.
